# Transient Overexpression of *HvSERK2* Improves Barley Resistance to Powdery Mildew

**DOI:** 10.3390/ijms19041226

**Published:** 2018-04-18

**Authors:** Yingbo Li, Qingwei Li, Guimei Guo, Ting He, Runhong Gao, Muhammad Faheem, Jianhua Huang, Ruiju Lu, Chenghong Liu

**Affiliations:** 1 Biotech Research Institute, Shanghai Academy of Agricultural Sciences/Key Laboratory of Agricultural Genetics and Breeding, Shanghai 201106, China; liyingbo163@163.com (Y.L.); 18739760975@163.com (Q.L.); guo_gm@126.com (G.G.); green1216@sina.com (T.H.); gaorunhong2005@163.com (R.G.); sw1@saas.sh.cn (J.H.); 2 Barani Agricultural Research Institute, Chakwal 48800, Pakistan; mlofaheem@gmail.com

**Keywords:** barley, SERK, powdery mildew, transient overexpression

## Abstract

Somatic embryogenesis receptor-like kinases (SERKs) play an essential role in plant response to pathogen infection. Here we identified three *SERK* genes (*HvSERK1/2/3*) from barley, and aimed to determine their implication in defense responses to barley powdery mildew (*Bgh*). Although *HvSERK1/2/3* share the characteristic domains of the SERK family, only *HvSERK2* was significantly induced in barley leaves during *Bgh* infection. The expression of *HvSERK2* was rapidly induced by hydrogen peroxide (H_2_O_2_) treatment, but not by treatment with salicylic acid (SA), methyl jasmonate (MeJA), ethephon (ETH), or abscisic acid (ABA). Bioinformatics analysis of the cloned *HvSERK2* promoter revealed that it contains several elements responsible for defense responses against pathogens. Promoter functional analysis showed that the *HvSERK2* promoter was induced by *Bgh* and H_2_O_2_. Subcellular localization analysis of *HvSERK2* indicated that it is mainly located on the plasma membrane. Transient overexpression of *HvSERK2* in epidermal cells of the susceptible barley cultivar Hua 30 reduced the *Bgh* haustorium index from 58.6% to 43.2%. This study suggests that the *HvSERK2* gene plays a positive role in the improvement of barley resistance to powdery mildew, and provides new insight into the function of *SERK* genes in the biotic stress response of plants.

## 1. Introduction

Plant diseases are responsible for billions of dollars of crop losses worldwide every year. Powdery mildew, caused by *Blumeria graminis* f. sp. *hordei* (*Bgh*), is one of the most serious diseases affecting barley yield worldwide. Powdery mildew is an obligate biotroph, the fungus is entirely restricted to epidermal leaf cells for the establishment of a haustorium; an essential organ that provides nutrition to the fungi [1]. Plants exhibit a wide array of defense strategies to defend themselves against attacks from various micro-organisms [2]. The first defense strategy of the plant immune system is PAMP-triggered immunity (PTI), which involves basal defense responses that are triggered upon detection of pathogen-associated molecular patterns (PAMPs) [3]. It is common for membrane proteins to format receptor complexes linking extracellular perception to intracellular signal transduction in the plant PTI response [3]. A typical response of PAMPs is the apoplastic accumulation of reactive oxygen species such as hydrogen peroxide (H_2_O_2_) and the superoxide anion [4]. In order to suppress PTI, pathogens secrete small unique protein molecules known as effectors. Upon recognition of the effectors, the host plant triggers the second defense line, effector triggered immunity (ETI), which always leads to cell apoptosis [5].

Receptor-like kinases (RLKs), which comprise a large gene family responsible for various signal transduction processes, proved to play a significant role in plant–pathogen interactions [6]. Among various *RLK* genes, somatic embryogenesis receptor kinases (SERKs) belong to the leucine-rich repeat receptor-like protein kinase (LRR-RLK) superfamily [7]. This gene family shares a N-terminus signal peptide (SP), an extracellular leucine zipper (LZ), five leucine rich repeats (LRRs) in its extracellular domain, a distinct serine–proline–proline (SPP) domain, a single TM (transmembrane) domain, a serine/threonine kinase domain, and a C-terminus region. As well as playing a major role in embryogenesis, SERKs have been shown to participate in the pathogen cell death response [8]. *Arabidopsis* SERK3/BAK1 forms a complex with BRI1 to ensure full brassinosteroid signaling, SERK3 also exists in a preformed complex with FLS2 to act as a positive PTI regulator [9,10,11]. The rice *SERK* gene *OsSERK1* was induced by defense signaling molecules such as salicylic acid (SA), jasmonic acid (JA), and abscisic acid (ABA), and was associated with host cell death, imparting resistance to the rice blast fungus [12]. *OsSERK2* can positively regulate the immune response to *Xanthomonas oryzae pv. oryzae*, the function was mediated by *XA21* and *XA3* [13]. However, it is unknown whether *SERK* genes positively regulate defense to powdery mildew caused by *Bgh*. To the best of our knowledge, the role of SERKs in the barley–powdery mildew interaction has not been investigated previously.

The single-cell transient overexpression assay (TOA) is a highly reliable, quick, and powerful approach to study genes during *Bgh* infection, as the fungus only penetrates one cell layer. The technique was first applied with co-expression of the *B-peru* gene and defense-related genes in barley coleoptile cells [14]. Shirasu et al. [15] improved the system by co-expressing the green fluorescent protein (*GFP*) gene and *Mlo* or *mlo* alleles in barley leaves. Schweizer et al. [16] bombarded young wheat leaves with tungsten particles coated with a β-glucuronidase (*GUS*) reporter gene and a test gene. Currently TOA is widely used for elucidating the function of genes involved in the powdery mildew resistance of wheat and barley [17].

In this study, we reported that the gene *HvSERK2* improved barley resistance to *Bgh* infection, which is verified by the results of the TOA. We identified three *SERK* genes (*HvSERK1/2/3*) from barley cultivar Hua 30, and characterized their expressions against *Bgh* induction. Given that the expression of *HvSERK2* was significantly induced upon *Bgh* infection, its subcellular localization and response to defense signaling molecules were further investigated. Our research provides new insight into the roles of the *SERK* gene family in the plant biotic defense response.

## 2. Results

### 2.1. Cloning and Identification of Three SERK Genes from Barley

**Figure 1:**
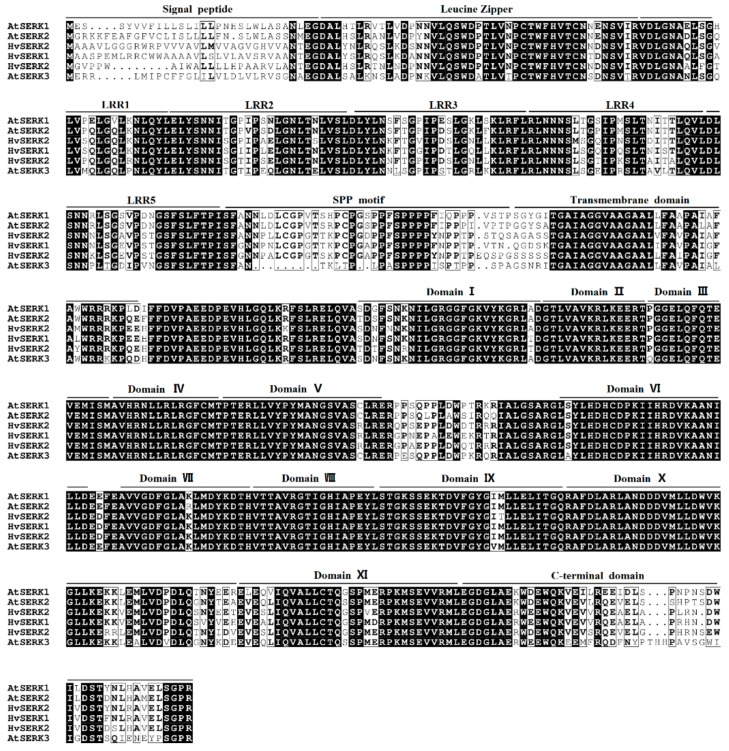
Comparison of three somatic embryogenesis receptor-like kinase (SERK) proteins from *Hordeum vulgare* L. and *Arabidopsis thaliana* SERK1, SERK2, and SERK3 proteins. Putative domains are indicated at the top of the sequences. The 11 subdomains of the protein kinase domain are marked with Roman numerals.

Three *SERK* genes were identified in the database (http://webblast.ipk-gatersleben.de/barley/) by a homology search for the SPP motif between the LRRs and the transmembrane domain (*HvSERK1*, GI: AK372118; *HvSERK2*, GI: AK252995; *HvSERK3*, GI: AK374641). We then homology cloned the three genes from the barley cultivar Hua 30. The amino acid sequences of three *SERK* genes were aligned with the three identified SERK proteins from *Arabidopsis thaliana*, indicating that the proteins shared the typical characteristic conserved domains of the SERK protein family, including five LRRs, a SPP motif, a TM domain, kinase domains, and a C-terminus domain (Figure 1).

**Figure 2:**
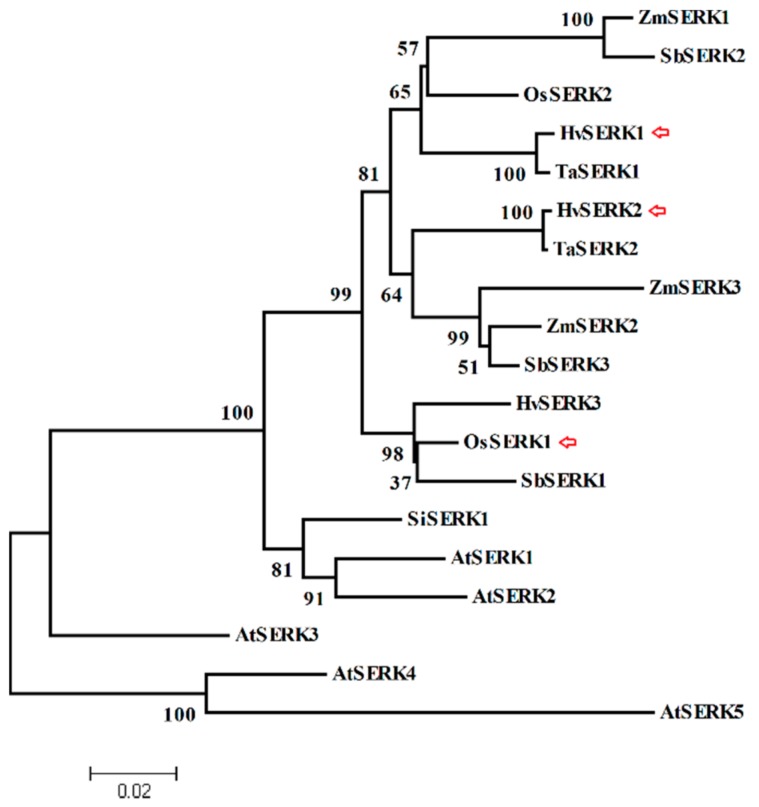
Phylogenetic analysis of the three barley SERK proteins and other plant SERK amino acid sequences. OsSERK1 (BAD05545.1), OsSERK2 (XP_015636497.1) from *Oryza sativa*; TaSERK1 (AEP14551.1) and TaSERK2 (AEP14552.1) from *Triticum aestivum*; ZmSERK1 (NP_001105132.1), ZmSERK2 (CAC37639.1), and ZmSERK3 (CAC37642.1) from *Zea mays*; SbSERK1 (EES13434.1), SbSERK2 (XP_002447957.1), and SbSERK3 (XP_002454054.1) from *Sorghum bicolor*; SlSERK1 (NP_001233866.1) from *Solanum lycopersicum*; AtSERK1 (NP_177328.1), AtSERK2 (AAK68073.1), AtSERK3 (AAK68074.1), At-SERK4 (NP_178999.2), and AtSERK5 (NP_179000.3)from *Arabidopsis thaliana*. The red arrows show SERK proteins from *Hordeum vulgare* L.

The phylogenetic relationships of the three barley *SERK* genes and other *SERK* homologs from different species were assessed by constructing a phylogenetic tree. As shown in Figure 2, the three barley *SERK* genes were located in different branches, which suggested that these genes have different functions during their evolution. Moreover, the *HvSERK1/2/3* cluster was similar to the SERK proteins of monocot species, indicating that the function of the three *SERK*-like genes might remain conserved in monocot species.

### 2.2. Characterization of the Response of Three SERK Genes to Bgh Infection

**Figure 3:**
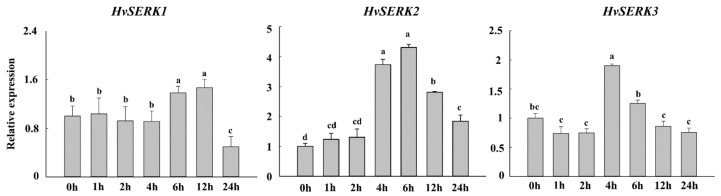
Expression profiles of *HvSERK1/2/3* in response to *Bgh* infection by qRT-PCR. The *actin* gene was used as an internal control to normalize qRT-PCR values. Different letters indicate statistically significant differences (*p* < 0.05, one-way ANOVA).

Three SERK gene expression profiles were investigated during *Bgh* infection in barley leaves. As shown in Figure 3, the expression profile of *HvSERK2* upon *Bgh* inoculation showed that *HvSERK2* was rapidly up-regulated (*p* < 0.05, ≥two-fold), peaked at 6 h post inoculation (hpi), remained stagnant for the next 6 h (12 hpi), thereafter the expression decreased to a normal level. However, there was no up- or down-regulation (*p* < 0.05, ≥two-fold) of the expression profile of the other two genes (*HvSERK1*/*HvSERK3*) after *Bgh* inoculation. This indicated that *HvSERK2* may function in the barley-*Bgh* interaction, so we selected *HvSERK2* for further investigation.

### 2.3. Characterization of HvSERK2 Responses to Defense Signal Molecules

**Figure 4:**
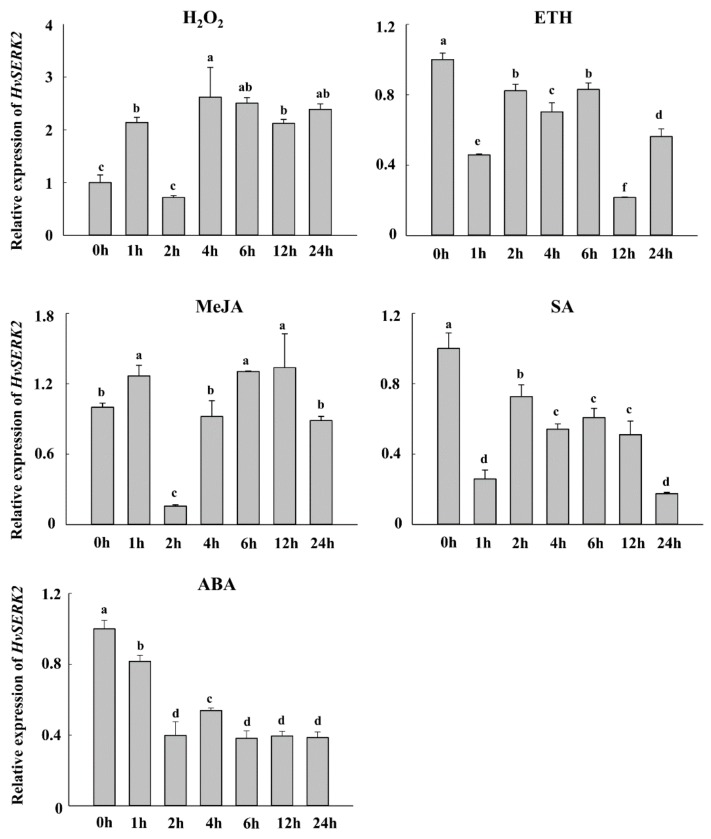
Expression profiles of *HvSERK2* in response to hydrogen peroxide (H_2_O_2_), ethephon (ETH), methyl jasmonate (MeJA), and salicylic (SA) treatments by quantitative RT-PCR. Expression profiles of *HvSERK2* in leaves of barley upon treatment with H_2_O_2_ (7 mM), ETH (200 mΜ), MeJA (0.1 mM), SA (5 mM), an dabscisic acid (ABA) (0.2 mM), respectively. The *actin* gene was used as an internal control to normalize qRT-PCR values. Different letters indicate statistically significant differences (*p* < 0.05, one-way ANOVA).

*HvSERK2* expression profiles were further investigated under treatment with different signaling molecules, which closely correlated to the disease resistance of the plant. After H_2_O_2_ treatment, a *HvSERK2* expression peak was first observed 1 h post-treatment (hpt), expression then decreased and peaked again 4 hpt, a high level of expression was maintained at the following detection time points (Figure 4). Upon both ethephon (ETH) and SA treatments, a slight decrease in the *HvSERK2* expression occurred 1 hpt, whereas, a decrease in *HvSERK2* expression was observed 2 hpt in response to methyl jasmonate (MeJA) and ABA application (Figure 4). These results indicated that *HvSERK2* expression in barley was induced by H_2_O_2_, and that *HvSERK2* might be involved in H_2_O_2_ signaling pathways in barley.

### 2.4. Analysis of the Cis-Regulatory Motifs for Cloned HvSERK2 Promoter

As shown in the above results, *HvSERK2* was induced by *Bgh* infection and H_2_O_2_ treatment (Figure 3 and Figure 4), therefore, we further cloned the *HvSERK2* promoter to investigate whether it contained motifs related to biotic or abiotic stress. By searching the available databases and conducting a bioinformatics analysis, we designed primers and cloned 2 kb of sequence upstream from *HvSERK2* (Supplementary Materials data 1). *In silico* analysis of the promoter of *HvSERK2* in Plant CARE and PsLACE revealed several elements responsible for defense or stress responses to pathogens (Supplementary Materials Table S1). Among the *cis*-acting regulatory elements found in the *HvSERK2* promoter, two fungal elicitor responsive elements, Box w1,were present on (−) strands at the positions of −325 bp and −961 bp, respectively. In addition, two motifs for MeJA responsiveness (TCT motif and CGTCA motif) and one TCA element for SA acid responsiveness were also annotated in the *HvSERK2* promoter. The sequence analysis also detected a 14 TATA-box (Supplementary Materials Table S1).

Detailed analysis of the promoter also revealed the presence of some key regulating motifs for abiotic stresses (Supplementary Materials Table S1). For example, an ABRE motif that related to abscisic acid responsiveness was found at a distance of −1388 bp from the initiation codon, and a HSE motif that related to heat stress responsiveness was found at a distance of −446 bp from the initiation codon. TC-rich repeats, which are involved in defense and stress responsiveness, were found −392 bp from the initiation codon. A total of 8 regulatory motifs (ACE, AE-box, Box 4, G-Box, GA-motif, MNF1, Sp1, and TCCC) that are involved in the regulation of the light responses of plants were found in the *HvSERK2* promoter. 21 CAAT box motifs were found on different strands (+ and −), and were found to enhance promoter activities (Supplementary Materials Table S1). These findings were partly consistent with *HvSERK2* expression during powdery mildew infection.

### 2.5. Functional Analysis of HvSERK2 Promoter

**Figure 5:**
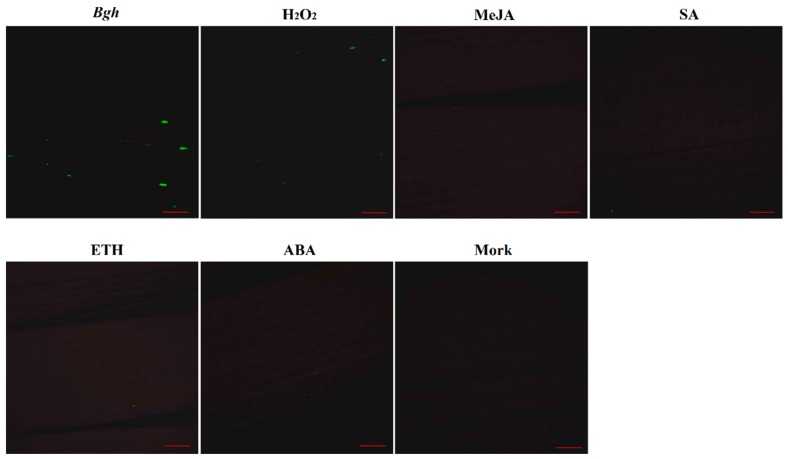
Activity of the *HvSERK2* promoter in response to *Bgh*, H_2_O_2_, MeJA, SA, ETH, and ABA treatments. Mork is control. Bars = 1 mm.

Based on *in silico* analysis of the *HvSERK2* promoter, we constructed a *pAN580:HvSERK2P:GFP* plasmid by replacing the CaMV 35S promoter of the *pAN580* vector with a *HvSERK2* promoter. The plasmid was transiently expressed in barley leaves by use of a gene gun. Bombarded leaves were treated with *Bgh*, H_2_O_2_, MeJA, SA, ETH, and ABA, respectively. As shown in Figure 5, bombarded leaves were inoculated with *Bgh* and H_2_O_2_, and could detect GFP signal, whereas, GFP signal could not be detected in bombarded leaves treated with MeJA, SA, ETH, and ABA. These results were consistent with the expression profile of *HvSERK2*, and indicated that *HvSERK2* is involved in the *Bgh* response and the H_2_O_2_ signaling pathway in barley.

### 2.6. Subcellular Localization of HvSERK2

**Figure 6:**
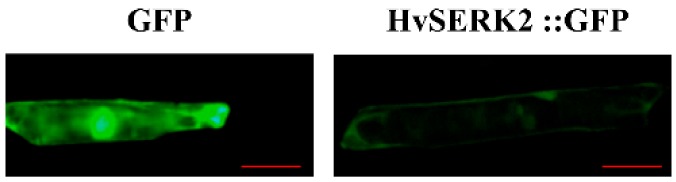
Subcellular localization of *HvSERK2* protein in onion epidermal cells. Bars = 100 μm.

To determine *HvSERK2* protein localization in plant cells, a construct containing the fusion product of the *HvSERK2* coding sequence and coding region of the green fluorescent protein (HvSERK2::GFP), under the control of the cauliflower mosaic virus (CaMV) 35S promoter, was transiently expressed in living onion epidermal cells. As shown in Figure 6, cells transiently expressing GFP (control) exhibited a uniform distribution of green fluorescence throughout the cell. In contrast, signal generated in the onion epidermal cells that transiently expressed HvSERK2::GFP was confined to the plasma membrane (Figure 6), thus, suggesting that *HvSERK2* was restricted to plasma membrane compartments.

### 2.7. Functional Analysis of HvSERK2 in Bgh Infection by TOA

**Figure 7:**
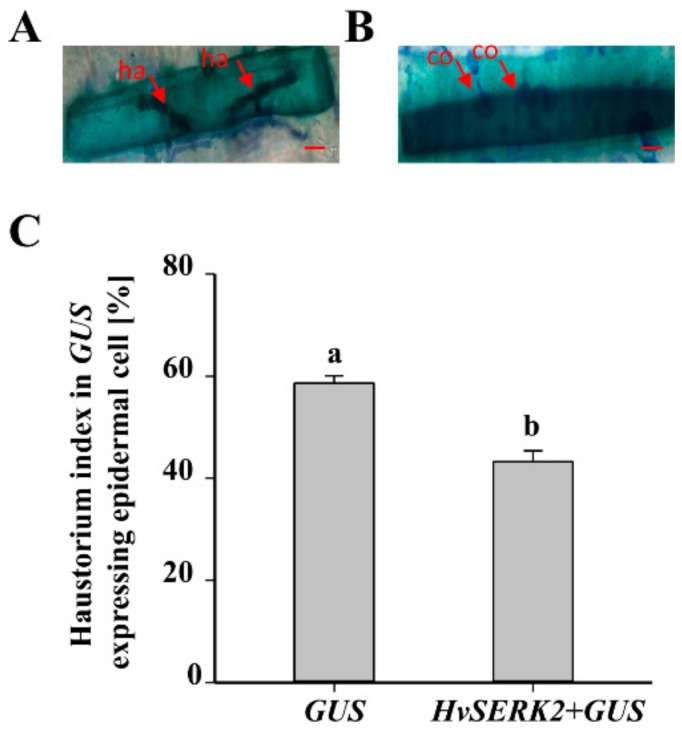
Functional analysis of *HvSERK2* by single-cell transient overexpression assay. (**A**) *Bgh* conidia successfully penetrated epidermal cells co-expressing *GUS* and *HvSERK2* with haustorium; (**B**) *Bgh* conidia failed to penetrate epidermal cells co-expressing *GUS* and *HvSERK2*. Bars in (**A**,**B**) = 20 μm; (**C**) in comparison with transforming *GUS* alone, the haustorium index of the cells co-transformed with *GUS* and *HvSERK2* significantly decreased. ha: haustorium; co: conidium. Bars with different letters show significant differences at the level of *p* < 0.05.

The haustorium index is usually used as a criterion to estimate the compatibility of interactions between the host and *Bgh*. To further characterize the role of *HvSERK2* in the powdery mildew response, TOA was conducted using the susceptible barley cultivar Hua 30 as a receptor. Comparing GUS-staining cells with haustoria to all GUS-stained cells invaded by *Bgh* (Figure 7A,B), the haustorium index (HI) for Hua 30 was 58.6% when transformed with *GUS* alone, but decreased significantly to 43.2% when co-transformed with *GUS* and *pBI220*-*HvSERK2* (Figure 7, Supplementary Materials Table S2). Whereas, when transformed with *pBI220*-*HvSERK1/3*, the HI for Hua 30 was 61.6% and 58%, respectively, compared to a HI for Hua 30 of 59.5% when transformed with *GUS* alone (Supplementary Materials Figure S1, Supplementary Materials Table S3). This indicated that the transient overexpression of *HvSERK2* prevented haustorium formation, so that the barley transform cells gained resistance to *Bgh*. This result demonstrated the involvement of *HvSERK2*, but not *HvSERK1/3*, in response to *Bgh* infection.

## 3. Discussion

The present study identified three SERK-like genes from barley, and characterized the function of the *HvSERK2* gene under *Bgh* infection. Typical SERK protein domain distribution is found in the three SERK proteins, phylogenetic analysis showed that the three barley SERK proteins were clustered closer to the SERK proteins from monocot species (*Oryza sativa*, *Triticum aestivum*, *Zea mays*, and *Sorghum bicolor*). This suggests that the SERKs of the grass family remained fairly conserved throughout their evolution. We also found that *HvSERK2* was clustered closer to the wheat SERK protein (TaSERK2) in the phylogenetic tree, but further away from the two other SERK proteins (*HvSERK1/3*) of barley. According to our results, only *HvSERK2* was induced by *Bgh* infection in barley leaves, which proved that *HvSERK2* had a diverse function when compared to the other two barley *SERK* genes. At the subcellular level, GmSERK1, from soybean, was localized to the plasma membrane [18], this was consistent with HvSERK2. This evidence suggests that *HvSERK2* functions as a LRR-RLK in the barley–powdery mildew interaction [19].

The typical model of LRR-RLK function states that extracellular domain binding of a signal molecule induces receptor dimerization, and then the intracellular kinase domain is activated by phosphorylation, which subsequently regulates the cellular response accordingly [20]. Depending on the system, the *SERK* gene family is not limited to somatic embryogenesis, and is involved in a diverse array of functions. Several elements, like abscisic acid responsiveness (ABRE), heat stress (HSE), TC-rich, and light responsive motifs (ACE, AE-box, Box 4, G-Box, GA-motif, MNF1, Sp1, and TCCC) were predicted in the in *HvSERK2* promoter, showing its involvement in different developmental metabolic process. A number of reports have been published on the role of SERKs in disease resistance responses. The *OsBISERK1* gene, isolated from rice, is reported to be involved in disease resistance responses and mediating defense signal transduction [21]. In *Arabidopsis*, the receptor kinase BAK1/SERK3 has been identified as a partner of brassinosteroid receptor BRI1 and immune receptor FLS2 [9]. The most important motif found that was related to pathogen defense in the *HvSERK2* promoter was the W-Box, which is recognized by WRKY DNA binding proteins that trigger the promoter and activate downstream *NPR1* genes for pathogen defense responses [22]. Functional analysis of the *HvSERK2* promoter showed that it was induced upon *Bgh* inoculation. This is consistent with the result that the expression of *HvSERK2* is induced by powdery mildew infection, suggesting that *HvSERK2* is involved in fungal pathogen resistance. In the TOA experiment, transient overexpression of *HvSERK*2 in barley leaf epidermal cells decreased the HI of *Bgh* which provided direct evidence that *HvSERK*2 positively mediates barley resistance to *Bgh*.

Phytohormones have been shown to be closely correlated with disease resistance by regulating pathogenesis-related (*PR*) genes or acting as early signaling components [23]. During the early stages of incompatible barley*Bgh* interactions, H_2_O_2_ has been reported to act as a diffusible signal for the induction of cellular protectant genes [24]. H_2_O_2_ either induces the hypersensitive reaction [25,26] and subsequent *PR* gene expression, [27] or acts directly as the defense substance [28]. Our results show that the expression of *HvSERK2* was quickly induced by exogenous H_2_O_2_ (2 hpt), this is consistent with the resulting functional analysis of the *HvSERK2* promoter upon H_2_O_2_ treatment. These results were not in agreement with *OsSERK1*, which was activated by SA, JA, and ABA [13], and unexpectedly, were not consistent with the *in silico* analysis of the *HvSERK2* promoter.

In conclusion, this study proposes that the *HvSERK2* gene plays a positive role in powdery mildew resistance in barley, possibly though the H_2_O_2_ signaling pathway.

## 4. Materials and Methods

### 4.1. Plant Materials, Growth Conditions and Treatment

Barley (*Hordeum vulgare* L.) cv. Hua 30 is a popular variety cultivated in the Yangtze River Delta of China. The barley seedlings were grown at 22–25 °C with a photoperiod of 12 h. Mixed races of *Bgh* were maintained on seedlings susceptible to powdery mildew (Hua 30), in a spore-proof greenhouse, under a 14 h light/10 h darkness (22/18 °C, 70% humidity) regime. Two-leaf stage seedlings were inoculated with *Bgh* or treated with exogenous hormones, including 5 mM SA, 0.1 μM MeJA, 200 mΜ ETH, 7 mM H_2_O_2_, and 0.2 mΜ ABA. All chemicals were administered in a 0.05% Tween 20 solution, with a 0.05% Tween 20 solution used as a mock treatment, all of the treatments followed Zhu et al. [29]. For gene expression analysis, leaves were harvested at different time intervals after different treatments (0 h, 1 h, 2 h, 4 h, 6 h, 12 h and 24 h). Three biological replicates were used for each assay.

### 4.2. Identification of Three SERK Genes in Barley

To identify *SERK* genes in barley, the amino acid sequences of reported SERK proteins in model plants including *Arabidopsis* (NP_177328.1, AAK68073.1, AAK68074.1, NP_178999.2, and NP_179000.3) and rice (BAD05545.1 and XP_015636497.1) were used to search in the International Barley Sequencing Consortium (http://webblast.ipk-gatersleben.de/barley/), and the National Centre for Biotechnology Information (NCBI) database (http://www.ncbi.nim.nih.gov/BLAST/nr/EST) using the TBLASTN program with a cut off E-value of 1 × 10^−10^. Three *SERK* genes (AK372118, AK252995 and AK374641) were found from the high-throughput sequencing of barley cv. Morex. The homology cloning of the ORF of *SERK* candidates from barley cultivar Hua 30 (cDNA) was carried out using specific primers (Supplementary Materials Table S4). For gene cloning, cDNA was used as template. PCR was performed in 25 μL using a 1× Taq DNA polymerase buffer, 0.8 mM/L MgCl_2_, 0.8 mM/L dNTPs, 100 mM/L primers, 1 unit of KOD-Taq (TOYOBO, Osaka, Japan) polymerase, and 50 ng Hua 30 cDNA as a template. The PCR was programmed for 95 °C 5 min for denaturation, followed by 29 cycles of 94 °C for 30 s, 59 °C for 45 s, and 68 °C for 2 min 30 s, and a final extension of 10 min at 68 °C. The PCR product was cloned using a p-easy cloning kit (TransGen Biotech, Beijing, China) and sequenced by the Thermo Fisher Scientific Corporation (Suzhou, China). The phylogenetic associations were inferred using the neighbor-joining method in MEGA 4.0 software (Tempe, AZ, USA) with SERK proteins from different species at 2000 bootstrap.

### 4.3. Cloning and In Silico Analysis of HvSERK2 Promoter

To search the putative promoter region of *HvSERK2* in barley, the complete ORF of *HvSERK2* was used for a homology search in the International Barley Sequencing Consortium (http://webblast.ipk-gatersleben.de/barley/). A 2 kb sequence upstream from Morex was found and used to design forward primers, while the only reverse primer was located in the ORF of *HvSERK2*. The list of all primers used in the study is given in the Supplementary Materials Table S4. For promoter analysis, the sequence was searched for *cis* regulatory elements in two plant promoter data bases *viz*. Plant CARE (http://bioinformatics.psb.ugent.be/webtools/plantcare/html/) and PLACE (http://www.dna.affrc.go.jp/htdocs/PLACE/).

### 4.4. DNA, RNA Extraction and Transcription Analysis

DNA was extracted from 5 g of leaves of Hua 30 seedlings using the Cetyltrimethyl Ammonium Bromide (CTAB) method. For promoter amplification, genomic DNA was used as the template. PCR conditions included initial denaturation at 94 °C for 5 min then 29 cycles (94 °C for 30 s, 57 °C for 45 s, and 68 °C for 2 min 30 s) followed by final elongation at 68 °C for 10 min. The PCR product was cloned using a p-easy cloning kit and sequenced by the Thermo Fisher Scientific Corporation.

Total RNA of each sample was extracted using TRIZOL reagent, according to the manufacturer’s protocol (Thermo Fisher Scientific Corporation). Two microgram aliquots of total RNA of each sample were used to synthesize the first-strand cDNA using a Prime Script™ II first strand cDNA synthesis kit (TAKARA, Dalian, China). For expression pattern analysis, specific pairs of primers were used, and the *actin* gene GI: AK356840.1 was used as the internal control for normalization. qRT-PCR was performed with a SYBR Green ABI 7500 fast detection system (Thermo Fisher Scientific Corporation): 95 °C for 1 min, 41 cycles of 95 °C for 10 s, and 60 °C for 31 s. For each sample, the *Ct* value of each target gene was normalized to the *Ct* value of the *actin* gene (Supplementary Materials Table S4). The relative value of gene expression was derived from 2^−^^∆∆*C*T^ [30]. Three independent biological replications were performed for each treatment. The significant differences in each treatment were analyzed by paired sample one-way ANOVA using SPSS software (International Business Machines Corporation, New York, NY, USA).

### 4.5. Subcellular Localization of HvSERK2

XbaI and SmaI sites were added to the 5′ and 3′ ends of the full length open reading frame of *HvSERK2*, respectively (Supplementary Materials Table S4), with the stop codon deleted, as per appropriate design of the HvSERK2-SmaI-R primer. The PCR product and the *pAN580* vector were digested by XbaI and SmaI, and the fragments were ligated to produce the fusion gene expression vector p35S::HvSERK2-GFP::Nos3. For transient expression in onion, the constructs were delivered to epidermal cells on the adaxial surfaces of tissue peeled from an onion by particle bombardment, as described by Faheem et al. [31]. GFP signals were assessed by OLYMPUS SZX16 (OLYMPUS, Tokyo, Japan) imaging 16–20 h after bombardment.

### 4.6. Single-Cell Transient Overexpression Assay

The TOA of *HvSERK1/2/3* was performed using a Bio-Rad He/1000 particle delivery system (Bio-Rad, Hercules, CA, USA) with barley leaves, according to Schweizer et al. [15]. In brief, *HvSERK1/2/3* was cloned into the plant expression vector *pBI220* [29] to produce a *pBI220:HvSERK1/2/3* plasmid, while under the regulation of the CaMV 35S promoter (Supplementary Materials Table S3). The reporter plasmid *pWMB002*, containing the β-glucuronidase (*GUS*) gene and the expression plasmid *pBI220: HvSERK1/2/3*, was mixed before coating of the particles (molar ratio of 1:1; 1 µg of total DNA). The second leaves of one-week-old barley seedlings were firmly placed on the 1% agar plates, which were supplemented with 85 µM of benzimidazoles for the delivery of mixed plasmids into host cells by gene gun. Delivery of *pWMB002* alone was used as control. Bombarded leaves were incubated at 22 °C for 4 to 6 h in darkness, then infected with fresh *Bgh* conidia spores and placed in a growth chamber with a 14 h light/10 h darkness photoperiod for 48 h. Leaves were stained to determine the GUS activity, then bleached with 95% ethanol, and observed under a microscope to record the haustorial index (HI, percentage of GUS-staining cells with haustoria among all GUS-stained cells invaded by *Bgh*). The assay was repeated thrice and significant differences between treatments were analyzed by paired sample *t*-tests using SPSS software.

### 4.7. Function Analysis of HvSERK2 Promoter

The *HvSERK2* promoter was cloned into plant expression vector pAN580 [31] by replacing the CaMV 35S promoter to produce a *pAN580:HvSERK2P:GFP* plasmid (Supplementary Materials Table S3). Second leaves of one-week-old barley seedlings were firmly placed on the 1% agar plates and were supplemented with 85 µM of benzimidazoles for the delivery of the *pAN580:HvSERK2P:GFP* plasmid into host cells by gene gun. Bombarded leaves were incubated at 22 °C for 4 to 6 h in darkness, then were inoculated with *Bgh* or treated with exogenous hormones, including 5 mM SA, 0.1 mΜ MeJA, 200 mΜ ETH, 7 mM H_2_O_2_, and 0.2 mΜ ABA. All chemicals were administered in a 0.05% Tween 20 solution, and a 0.05% Tween 20 solution was used as a mock treatment. All treatments followed the instructions outlined by Zhu et al. [29]; solutions were placed in a darkness growth chamber after treatment. GFP signals were assessed by OLYMPUS SZX16 imaging 16–20 h after bombardment.

## Supplementary Materials

Supplementary materials can be found at http://www.mdpi.com/1422-0067/19/4/1226/s1.

## Abbreviations

SERK	Somatic embryogenesis receptor-like kinase
Bgh	*Blumeria graminis* f. Sp. *hordei*
PAMP	Pathogen-associated molecular patterns
PTI	PAMP-triggered immunity
ETI	Effector-triggered immunity
RLKs	Receptor-like kinases
LZ	Leucine zipper
LRRs	Leucine rich repeats
SPP	Serine proline proline
BR	Brassinosteroid
Xoo	*Xanthomonas oryzae pv. Oryzae*
TOA	Transient overexpression assay
MeJA	Methyl jasmonate
SA	Salicylic acid
H_2_O_2_	Hydrogen peroxide
ETH	Ethephon
ABA	Abscisic acid
hpt	Hours post-treatment
hpi	Hours post-inoculation

## References

[1] Zhang Z., Henderson C., Perfect E., Carver T.L.W., Thomas B.J., Skamnioti P., Gurr S.J. (2005). Of genes and genomes, needles and haystacks: *Blumeria graminis* and functionality. Mol. Plant Pathol..

[2] Dangl J.L., Dietrich R.A., Richberg M.H. (1996). Death don’t have no mercy: Cell death programs in plant-microbe interactions. Plant Cell.

[3] Zipfel C. (2009). Early molecular events in PAMP-triggered immunity. Curr. Opin. Plant Boil..

[4] Chisholm S.T., Coaker G., Day B., Staskawicz B.J. (2006). Host-microbe interactions: Shaping the evolution of the plant immune response. Cell.

[5] Nicaise V., Roux M., Zipfel C. (2009). Recent advances in PAMP-triggered immunity against bacteria: Pattern recognition receptors watch over and raise the alarm. Plant Physiol..

[6] Zipfel C. (2014). Plant pattern-recognition receptors. Trends Immunol..

[7] Li J. (2010). Multi-tasking of somatic embryogenesis receptor-like protein kinases. Curr. Opin. Plant Biol..

[8] Santos M.O., Aragão F.J. (2009). Role of *SERK* genes in plant environmental response. Plant Signal. Behav..

[9] Heese A., Hann D.R., Gimenez-Ibanez S., Jones A.M.E., He K., Li J., Schroeder I.J., Peck S.C., Rathjen J.P. (2007). The receptor-like kinase SERK3/BAK1 is a central regulator of innate immunity in plants. Proc. Natl. Acad. Sci. USA.

[10] Clouse S.D. (2011). Brassinosteroid signal transduction: From receptor kinase activation to transcriptional networks regulating plant development. Plant Cell.

[11] He K., Gou X., Yuan T., Lin H., Asami T., Yoshida S., Russell1 S.D., Li J. (2007). BAK1 and BKK1 regulate brassinosteroid-dependent growth and brassinosteroid-independent cell-death pathways. Curr. Biol..

[12] Hu H., Xiong L., Yang Y. (2005). Rice *SERK1* gene positively regulates somatic embryogenesis of cultured cell and host defense response against fungal infection. Planta.

[13] Chen X., Zuo S., Schwessinger B., Chern M., Canlas P.E., Ruan D., Zhou X., Wang J., Daudi A., Petzold C.J. (2014). An XA21-associated kinase (OsSERK2) regulates immunity mediated by the XA21 and XA3 immune receptors. Mol. Plant.

[14] Nelson A.J., Bushnell W.R. (1997). Transient expression of an thocyanin genes in barley epidermal cells: Potential for use in evaluation of disease response genes. Transgenic Res..

[15] Shirasu K., Nielsen K., Piffanelli P., Oliver R., Schulze-Lefert P. (1999). Cell-autonomous complementation of *mlo* resistance using a biolistic transient expression system. Plant J..

[16] Schweizer P., Pokorny J., Abderhalden O., Dudler R. (1999). A transient assay system for the functional assessment of defense-related genes in wheat. Mol. Plant-Microbe Interact..

[17] Rajaraman J., Douchkov D., Hensel G., Stefanato F.L., Gordon A., Ereful N., Caldararu O.F., Petrescu A.J., Kumlehn J., Boyd L.A. (2016). An LRR/malectin receptor-like kinase mediates resistance to non-adapted and adapted powdery mildew fungi in barley and wheat. Front. Plant Sci..

[18] Yang C., Zhao T., Yu D., Gai J. (2011). Isolation and functional characterization of a *SERK* gene from soybean (*Glycine max* (L.) Merr.). Plant Mol. Biol. Rep..

[19] Walker J.C. (1994). Structure and function of the receptor-like protein kinases of higher plants. Plant Mol. Biol..

[20] Becraft P.W. (2002). Receptor kinase signaling in plant development. Annu. Rev. Cell Dev. Biol..

[21] Song D., Li G., Song F., Zheng Z. (2008). Molecular characterization and expression analysis of OsBISERK1, a gene encoding a leucine rich repeat receptor-like kinase, during disease resistance responses in rice. Mol. Biol. Rep..

[22] Yu D., Chen C., Chen Z. (2001). Evidence for an important role of WRKY DNA binding proteins in the regulation of *NPR1* gene expression. Plant Cell.

[23] Bari R., Jones J.D. (2009). Role of plant hormones in plant defence responses. Plant Mol. Biol..

[24] Vanacker H., Carver T.L., Foyer C.H. (2000). Early H_2_O_2_ accumulation in mesophyll cells leads to induction of glutathione during the hypersensitive response in the barley powdery mildew interaction. Plant Physiol..

[25] Tenhaken R., Levine A., Brisson L.F., Dixon R.A., Lamb C. (1995). Function of the oxidative burst in hypersensitive disease resistance. Proc. Natl. Acad. Sci. USA.

[26] Torres M.A., Jones J.D.G., Dangl J.L. (2005). Pathogen-induced, NADPH oxidase-derived reactive oxygen intermediates suppress spread of cell death in *Arabidopsis thaliana*. Nat. Genet..

[27] Alvarez M.E., Pennell R.I., Meijer P.J., Ishikawa A., Dixon R.A., Lamb C. (1998). Reactive oxygen intermediates mediate a systemic signal network in the establishment of plant immunity. Cell.

[28] Baker C.J., Orlandi E.W. (1995). Active oxygen in plant pathogenesis. Annu. Rev. Phytopathol..

[29] Zhu Y., Li Y., Fei F., Wang Z., Wang W., Cao A., Liu Y., Han S., Xing L., Wang H. (2015). An E3 ubiquitin ligase gene *CMPG1-V* from *Haynaldia villosa* L. contributes to the powdery mildew resistance in common wheat. Plant J..

[30] Livak K.J., Schmittgen T.D. (2001). Analysis of relative gene expression data using real-time quantitative PCR and the 2^−ΔΔ*C*T^ method. Methods.

[31] Faheem M., Li Y., Arshad M., Cheng J., Zhao J., Wang Z., Xiao J., Wang H., Cao A., Xing L. (2016). A disulphide isomerase gene (PDI-V) from *Haynaldia villosa* contributes to powdery mildew resistance in common wheat. Sci. Rep..

