# Supplementary material for: Transient Overexpression of HvSERK2 Improves Barley Resistance to Powdery Mildew
Source: Int J Mol Sci. 2018 Apr 18;19(4):1226. doi: 10.3390/ijms19041226 (PMC5979413; doi:10.3390/ijms19041226)
Supplement: Supplementary file 1 [file ijms-19-01226-s001.pdf]

Supplementary Materials:

Supplementary data 1. Sequence of *HvSERK2* promoter

CCTTAACAGTGCCCATATTCGGCGGGTCACTTAACAAGCAATATCCCCAACAGGTAGGGTTTGTCTGG
CGGCCCGTGTGAGAGTGGGACGAGGCGAGCACGGGATTGGTTGCAAGACGCGCGGAGCAGATTGGC
CAAAAACCTAGTGCACGCGGGAGTCGACGAACCCAGCCAACCAACATGCAACGTGAGCCCACCTGG
CGAGCAAAAACAACCGCAGGATGAAAATCAAGTTTAACTGTTGTAAAGATCTAACGACTCAGACACA
TGAAAAGGCTAATCGGACGGTCAGAGAAACCCCAAATCGGGGGAGCCCTTGGGAGGGCGGGTCGCC
CAACATCCTTAGGGCATGTACATCGGTTTAGACAATGTCTGACTATAATGGCTTGACATGTCATGTGCA
GACAACGCTAAGGGCAACATGTACATTGGGACCTTGTTTGGTCGGCTATTGAAACAAAAAACAATTTG
ACAAATCTTTTACTTGATCTAGACTTAACATTTGGGGATTTGATCGTGGCTGCTGAACTTACGCTGGAT
GGAAAGGCTAGACCGCTAGATCGACCACGGAAGGAGAAAACCGTTGTAATTGCAGACGGCCTCGATA
CAACGGTACATTGTCGTCTGTATCTCCCGATTTTCCTGCCCCGCAGATCGTCTGGTATTTTACCGACGAC
CTCTTTCCCTCTCTTGATTCTCTCTCCTCCACATCATTTAAAATCCTAATTATCAACTCTTATAGACAGCG
ACAGCTGATTGTACATCATTGTACATGCCCTTATAGGTTAGAATTGGCCCAAGTCCATGGGACCCACA
GCCCACTAACATCTGGCCTCATAGGACATTCTTTATTCAAAGCTCTCTGAAATCCCCGTAAATAAAAG
GGAGCTGCTAGGCGTCCACCGGTGGATCCGTATCAAATATCCATCACCCAAATAGCCGTCTGATTACG
CGCGCGTAGCCGTCCGATCTACCAGTTGGGTCGCTTCTTTCGGTCAAATACAACCTGAAACAACAACC
CAGTTGCGGAAACAATTGCTTTGTTGCGAAATAATTTTGGACCCATTAATTCTTGAAACAACAGTCGA
GTTGCAGAAACAATTGCTTTGTTGCAGAATTTTTTTCTTCGTCTTTGTACAACATAGGTACTGTTGCGGA
AGAATTTTTTGCAACAAAGATCATGTTGCAGAAAACTTTTACAACATAGATCAGCTTGTATAATTTTTT
GTCTTCATCTTTCTGCAACATAGATACTGTTGCGGAAGAAATTGTTGTAACATAGGTACTTTTGCAACT
GGCCTCAAGGGATCCAGATTCAGATCCTGCAACATAGGAGGTGCTGTGGTGCGCAAACTGCAACAAG
CGCTAAGTTGCACAAGTGCAAGCTAGGTGAGTCTACATAAAACAAGCGTTGCACAAGCGAGAGGCTC
TGCCCTTAAGGAATTCAGATTCAGATCCTGCATCATAGAACGTGATGCGGTACGCAATCTGCAACAAG
CGTTAAGTTACAGAATGATAAACTATACGAGTTCAAATAAGACACGCGTCGCACGGACGAGACGGCG
GCCGCACGATCAGCCGGCTGGAGCACAAGGTTTTCCTAAATAAAAAGAAAAACTTCGTTCACAAGGT
CAAAACAATGATTTTGCTCTACGGGCGACGAGAGTGGGCACAAAGACCGCGTCCACGAGATCCTCGG
CACACCGTCAGCAGATGCCCTCCTCCTGCTCCCTCCGTATTCTTCTCACTAGCTCCATCAATTCAGCCG
CCCCACCACACCCCCTGTACTCCTTCACCACCACACCTCATCGCTTTCATGTGGGCCCTCAACTCCGTG
ACCCACAAATCGGCCAAGTTGTAAGATTCCTGTAAGCTGTAGCCTTCCTCCTCACCCAGCATAGGCGA
TGGTCCCGTCGCCCAGTAGAACAATTTACTATACAAGTCGGAGCATACACACACGCACTACGAACTTA
ATTAAAGCTACAGAAATTTATCCCACCCATTCCATTAGGGAAGAGAGGGAGGGAGGAAGAAAGAAG
ACGCCGAGCTATCAACGGCGACGCGGCGGCCCAGCAGATCCGTACAGCTCGGGGGATCTTGGGGAAG
GGAGAACTGGTGGGGGCCGCCTATGCGTCGGTGAGGTGGGGCCCAGTCGGCGCTAGAGCAGGA

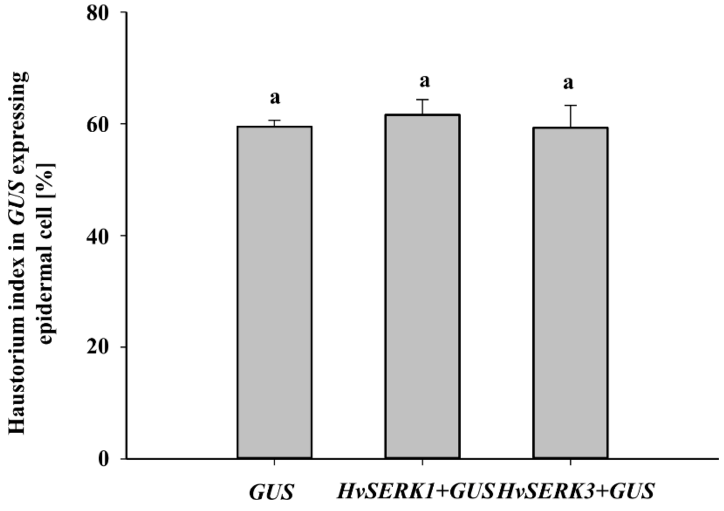

Supplementary Figure S1. Functional analysis of *HvSERK2* by single-cell transient overexpression assay. Bars with different letters show significant differences at the level of $p < 0.05$.

Supplementary Table S1. Key regulatory motifs found within the promoter sequence of *HvSERK2*

Regulatory Motif	Function	Position	Consensus Sequence
A-box	cis-acting regulatory element	+293	CCGTCC
ABRE	cis-acting element involved in the abscisic acid responsiveness	-1388	GCCGCGTGGC
ACE	cis-acting element involved in light responsiveness	+521	AAAACGTTTA
AE-box	part of a module for light response	+425	AGAAACAA
Box 4	part of a conserved DNA module involved in light responsiveness	+396	ATTAAT
Box-W1	fungal elicitor responsive element	-325, -961	TTGACC
CCAAT-box	MYBHv1 binding site	+1383	CAACGG
CCGTCC-box	cis-acting regulatory element related to meristem specific activation	+293	CCGTCC
CE3	cis-acting element involved in ABA and VP1 responsiveness	-870	GACGCGTGTC
CGTCA-motif	cis-acting regulatory element involved in the MeJA-responsiveness	+1036	CGTCA
G-Box	cis-acting regulatory element involved in light responsiveness	-800	CACGTT
GA-motif	part of a light responsive element	-563	AAAGATGA
GC-motif	enhancer-like element involved in anoxic specific inducibility	-1420	CCCCCG
HSE	cis-acting element involved in heat stress responsiveness	-446	AAAAAATTTC
LTR	cis-acting element involved in low-temperature responsiveness	-321	CCGAAA
MBS	MYB binding site involved in drought-inducibility	-360, -352, +324, +623	CAACTG
MNF1	light responsive element	-997	GTGCCC(A/T)(A/T)
O2-site	cis-acting regulatory element involved in zein metabolism regulation	-37	GATGATGTGG
Sp1	light responsive element	-1097, -1351, +1327, -1355	GGGCGG
TATC-box	cis-acting element involved in gibberellin-responsiveness	+1323	TATCCCA
TC-rich repeats	cis-acting element involved in defense and stress responsiveness	-836	ATTCTCTAAC
TCA-element	cis-acting element involved in salicylic acid responsiveness	-1068	GAGAAGAATA
TCCC-motif	part of a light responsive element	-1436	TCTCCCT
TCT-motif	part of a light responsive element	-1189	TCTTAC
TGACG-motif	cis-acting regulatory element involved in the MeJA-responsiveness	-1036	TGACG
Circadian	cis-acting regulatory element involved in circadian control	-508, -538, -581	CAANNNNATC
		Strand + (Number of motifs)	Strand - (Number of motifs)
CAAT box	cis-acting element in promoter and enhancer regions	11	10
TATA-box	core promoter element around -30 of transcription start	5	9

"+" downstream from the promoter sequence;"-" upstream from the start codon (ATG)

Supplementary Table S2. Functional analysis of the *HvSERK2* by single cell transient over-expression assay.

Genes		Total No. of observed cells	Observed cells with haustorium	Horstorium Index (%)	*t*-test (p < 0.05)
Replicate 1	*GUS*	84	48	57.1%	
	GUS+HvSERK2	78	32	41%	
Replicate 2	*GUS*	80	47	58.8%	
	GUS+ HvSERK2	60	26	43.3%	
Replicate 3	*GUS*	60	36	60%	
	GUS+ HvSERK2	75	34	45.3%	
Total	*GUS*	224	131	58.6%	a
	GUS+ HvSERK2	213	92	43.2%	b

Supplementary Table S3. Functional analysis of the *HvSERK1* and *HvSERK3* by single cell transient over-expression assay.

Genes		Total No. of observed cells	Observed cells with haustorium	Horstorium Index (%)	*t*-test (p<0.05)
Replicate 1	*GUS*	91	53	58.2%	
	GUS+HvSERK1	85	55	64.7%	
	GUS+HvSERK3	76	43	56.6%	
Replicate 2	*GUS*	103	62	60.2%	
	GUS+ HvSERK1	66	40	60.6%	
	GUS+HvSERK3	73	42	57.5%	
Replicate 3	*GUS*	83	50	60.2%	
	GUS+ HvSERK1	79	47	59.5%	
	GUS+HvSERK3	61	39	63.9%	
Total	*GUS*	277	165	59.5%	a
	GUS+ HvSERK1	230	142	61.6%	a
	GUS+HvSERK3	210	124	59.3%	a

Supplementary Table S4. Information of the primer pairs used in this study

Primer name	Primer sequence	Note
HvSERK1-F<br>HvSERK1-R	F: CGCTCGGATGGCTGCGTCGCCGG<br>R: TTTTCCTGTTACCTCGGGCCGGACAG	Cloning primer for *HvSERK1*
HvSERK2-F<br>HvSERK2-R	F: GAGCAGGAATGGCGGCGGCGGTGTTGGG<br>R: AATGATGTTTGTCATCTTGGGCCAGAC	Cloning primer for *HvSERK2*
HvSERK3-F<br>HvSERK3-R	F: ATCTAGGGCGGCGGCAATGGGGGT<br>R: AGCAGCCATCATCACCTTGGCCCT	Cloning primer for *HvSERK3*
HvSERK1-Q-F<br>HvSERK1-Q-R	F: ATGGCAGTGCATAGGAACCT<br>R: CAACCGCCTCAAAGTCTTCA	qRT-PCR primer for *HvSERK1*
HvSERK2-Q-F<br>HvSERK2-Q-R	F: GATGCTCTGTATAACCTGCG<br>R:TGACCAGGCTAGTTAGGTTG	qRT-PCR primer for *HvSERK2*
HvSERK3-Q-F<br>HvSERK3-Q-R	F: TCGGGCACAATTCCTAAATC<br>R: CCTGGTGATTGCTCAGGAGT	qRT-PCR primer for *HvSERK3*
Actin-F<br>Actin -R	F: GACTCTGGTGATGGTGTCAGC<br>R: GGCTGGAAGAGGACCTCAGG	qRT-PCR primer for *Actin*
HvSERK2-XbaI-F<br>HvSERK2-SmaI-R	F: GCTCTAGAATGGCGGCGGCGGTGTTGGGGG<br>R: TCCCCCGGGTCTTGGGCCAGACAGTTCCACT	Subcellular localization primer of *HvSERK2*
HvSERK2-KpnI-F<br>HvSERK2-SacI-F	F: GGGGTACCATGGCTGCGTCGCCGGA<br>R: CGAGCTCTTACCTCGGGCCGGACA	Transient over expression primer of *HvSERK1*
HvSERK2-KpnI-F<br>HvSERK2-SacI-R	F: GGGGTACCATGGCGGCGGCGGTGT<br>R: CGAGCTCTCATCTTGGGCCAGACA	Transient over expression primer of *HvSERK2*
HvSERK2-KpnI-F<br>HvSERK2-SmaI-R	F: GGGGTACCATGGGGGTGCCGCCGT<br>R: TCCCCCGGGTCACCTTGGCCCTGAT	Transient over expression primer of *HvSERK3*
HvSERK2P-F<br>HvSERK2P-R	F: CCTTAACAGTGCCCATATTCGG<br>R: AACAACCATCAACACAGCGAC	Cloning primer for *HvSERK2*promoter
HvSERK2P-SacI-F<br>HvSERK2P-SpeI-R	F: CGAGCTCCCTTAACAGTGCCCATATTCGG<br>R: GGACTAGTTCCTGCTCTAGCGCCGACTGGG	Activity analysis primer for *HvSERK2* promoter
